# Feeding System Effects on Dairy Cow Rumen Function and Milk Production

**DOI:** 10.3390/ani12040523

**Published:** 2022-02-21

**Authors:** Stephen McAuliffe, John F. Mee, Eva Lewis, Norann Galvin, Deirdre Hennessy

**Affiliations:** 1 Teagasc, Animal & Grassland Research and Innovation Centre, Moorepark, Fermoy, P61 P302 Cork, Ireland; stephenmcauliffe15@gmail.com (S.M.); john.mee@teagasc.ie (J.F.M.); norann.galvin@teagasc.ie (N.G.); 2 Institute of Global Food Security, Queen’s University Belfast, Belfast BT1 3BG, UK; 3 Devenish, Lagan House, 19 Clarendon Road, Belfast BT1 3BG, UK; eva.lewis@devenish.com

**Keywords:** clover, rumen, ammonia, volatile fatty acid, milk urea nitrogen, dairy cows

## Abstract

**Simple Summary:**

In ruminants, diet has a significant effect on rumen function. Hence, this study was conducted to see if changing the normal grass-based diet of cows could change rumen function and milk production. Therefore, we compared three diets: Grass (cows grazing grass-only), Grass-Clover (cows grazing grass-white clover only) and cows fed a total mixed ration (TMR) diet indoors. We monitored the cows over the course of a full lactation cycle in a spring-calving system. In order to examine rumen function, some of the cows had a cannula placed in their rumen so that we could collect rumen contents samples. After we collected the samples and analysed the data we found that the type of diet the cows were offered did significantly affect milk production; milk yield and milk solids yield were generally highest on the TMR diet. When we looked at the rumen sample data we found that diet also affected rumen function; it altered the rumen volatile fatty acid (VFA), and ammonia and lactic acid profiles significantly but did not effect on rumen pH. Clover inclusion in the diet led to higher total rumen VFA and ammonia concentrations and higher milk urea nitrogen compared to the grass and TMR diets. We suggest this indicates a higher protein intake on that diet. Given these findings, we concluded that a clover-based diet could significantly alter rumen function, milk composition and milk yield in dairy cows.

**Abstract:**

Good rumen function, which is largely influenced by the diet of the cow, is essential to optimise animal performance. This study, conducted over the course of a full lactation in a spring-calving milk production system, compared the rumen function and milk production of cows offered one of three dietary treatments: (1) Cows grazing grass-only swards receiving 250 kg nitrogen (N)/ha/year (Grass), (2) Cows grazing grass-white clover swards receiving 250 kg N/ha/year (Grass-Clover), and (3) Cows offered a total mixed ration diet and housed indoors (TMR). Treatment significantly affected milk production; milk yield and milk solids yield were generally highest on the TMR treatment. There was no effect of treatment on rumen pH. However, treatment significantly altered the rumen volatile fatty acid (VFA), and ammonia and lactic acid profiles. Clover inclusion in the sward led to higher (*p* < 0.05) total VFA and ammonia concentrations compared to the Grass and TMR treatments. The increased rumen ammonia concentration was associated with a significantly greater milk urea nitrogen (MUN) content in the milk from cows fed on Grass-Clover, indicating a greater excess of dietary protein in that treatment. It was concluded that a clover-based dairy cow feeding system could significantly alter rumen function, milk composition and milk yield.

## 1. Introduction

The introduction of the European milk quota scheme in 1984 resulted in quotas being the primary limiting factor of milk production on dairy farms in the EU. European dairy farmers have increased milk production since quotas were removed, as predicted [1]. With land now set to become the limiting factor of production on most farms [2], any increase in milk production will have to come from more efficient use of the current land area, the introduction of more land into the system, or through an increased use of supplementary feed and forage from outside the milking block.

Feed system/diet has a significant effect on milk and milk solids yield in any production system [3]. The primary challenge in feeding dairy cows is to provide an energetically high-density diet without compromising the ruminal ecosystem, animal welfare, and production performance [4]. Previous research has shown the potential of a well-balanced total mixed ration (TMR) diet to maximise milk production per cow [3,5,6]. Feeding TMR diets allows the diet to be altered to suit the cows’ requirements (age, stage of lactation, etc.), giving TMR an advantage over pasture-based systems in terms of attaining maximum output per cow [5,7]. It also greatly reduces the exposure of the herd to varying weather conditions and herbage quality while also being able to maintain a consistent diet.

In temperate regions, where grass grows over a long growing season such as Ireland, New Zealand, parts of the UK and north-west Europe, implementing TMR systems does not allow for the exploitation the ability of these regions to grow large quantities of grazed grass, which is a low-cost feed. Pasture-based systems are considered the most advantageous systems of milk production for Irish dairy farmers from an economic perspective given the lower capital investment required along with lower production costs; all of which help to insulate the system somewhat from volatile milk prices [8]. 

White clover (*Trifolium repens* L.; clover) inclusion in the sward has been shown to improve both pasture quality and animal performance compared to a grass-only sward [9,10]. This, combined with the ability of clover to fix up to 150 kg nitrogen (N)/ha/year [11], has led to increased interest in incorporating clover into grass pastures for high stocking rate systems (<2 cows/ha). However, the effect of clover inclusion in the diet on rumen function and possible subsequent environmental effects is poorly understood, particularly compared to TMR feeding systems. Differences in rumen characteristics, such as the quantity and proportions of volatile fatty acids (VFA) and rumen pH, have been reported between animals fed clover [12,13] or mixed swards [14] compared to perennial ryegrass only (grazing, zero-grazing grass or silage). However, these studies were either short term [14], used grass and grass clover silages rather than grazed swards [12] or focused on steer rather than dairy cow production [13]. Despite the importance of grass and grass-clover-based systems of milk production in temperate regions, little work has been completed, particularly in the recent past, in assessing the effect of these diets on the function of the rumen of the dairy cow. Specifically, while previous studies have compared grass-only to grass-clover diets [9] or grass-only to TMR diets [5], little if any work has been carried out simultaneously comparing both sward types to a TMR system. In addition. much improved grassland management techniques, which further optimise animal performance from grazed grass, have been developed over the past decade which warrant updated research to compare grazing and TMR systems of milk production. Thus, the objective of this study was to determine the effects of Grass, Grass-Clover and TMR feeding treatments on dairy cow milk production and rumen function. 

## 2. Materials and Methods

The experiment was undertaken at Teagasc, Animal and Grassland Research and Innovation Centre (Moorepark, Fermoy, Co. Cork, Ireland; 520 9′ N; 80 16′ W) on a free-draining, acid brown earth of sandy loam to loam texture. The 10-year average annual rainfall at the site (2007–2016 inclusive) was 1018 mm/year. This experiment was part of a larger experiment described previously [15]. 

### 2.1. Experimental Design and Animal Ethics

The study was undertaken over the course of one complete lactation (calving in February/March and dry-off in November). The experiment had three treatments: 1. Grass; cows grazing grass-only swards receiving 250 kg N/ha/year, 2. Grass-Clover; cows grazing grass-white clover swards receiving 250 kg N/ha/year and 3. TMR; cows offered a total mixed ration diet and housed indoors all year round. Soils had a pH of 6.4 and were index 3 and 4 for phosphorus (P) and potassium (K) and therefore no P or K fertilizer was applied. All experimental procedures involving cows were approved by the Teagasc Animal Ethics Committee and authorized by the Health Products Regulatory Authority (license AE19132-P019), which is the competent authority in Ireland responsible for the implementation of European Union legislation (Directive 2010/63/EU) for the protection of animals used for scientific purposes. 

There were three seasons (spring, summer and autumn (which were approximately early, mid- and late lactation, respectively)). Within each season a 3 (treatments) × 3 (periods) Latin square study was completed. Each period in the Latin square lasted two weeks, during which the first 10 days were for acclimatization and measurements were undertaken in the last four days. There were nine primiparous rumen-cannulated cows used, three per treatment per period. The cannulated cows were added to the grazing herds and the TMR herd for each season and were removed from those herds between seasons. Between seasons the cannulated cows were removed from the experimental area to non-experimental grassland area and grazed predominantly perennial ryegrass pasture. Each treatment herd has 17 “intact” cows.

The rumen-cannulated (101.6 mm internal diameter, Bar Diamond Inc., Parma, ID, USA) cows were blocked according to calving date (mean 7 February 2016 ± 6.7 days), body weight (447 ± 31 kg), body condition score [16] (2.96 ± 0.13), milk yield (19.3 ± 2.37 kg/cow per day) and milk solids yield (1.49 ± 0.18 kg/cow per day) for the three week period prior to commencement of the experiment. At the beginning of the experiment the cows were randomly assigned to one of the three treatments described above and then, as per the Latin Square design, they moved from one treatment to the next to the last. Rumen VFA, ammonia, lactic acid and pH data are from the rumen-cannulated cows. The milk production results are from the herd fed each diet during the three seasons—spring (April/May), summer (June/July) and autumn (August/September). 

### 2.2. Rumen Sampling and Rumen Contents Analyses

Rumen samples for individual analysis were collected from each of the cannulated cows after AM and PM milking on days 11 and 12 of each period. Liquid and solid rumen contents were taken from at least for locations in the rumen of each cow and strained through three layers of synthetic cheese cloth to separate the solids from the liquids. All samples were stored at −20 °C until analysis. Prior to analysis, the samples were thawed and centrifuged. The liquid fraction was used for VFA, ammonia and lactic acid (LA) analyses. For VFA and ammonia analysis, 2 mL of 50% trichloroacetic acid solution were added to 8 mL of rumen fluid. A 10 mL strained rumen fluid sample was used for L-lactic (L-LA) and D-lactic (D-LA) acid analysis. For the VFA estimation, 250 µL of the centrifuged liquid was mixed with 3.75 mL distilled water and 1 mL of a solution of 0.5 g of 3-methyl-N-valeric acid in 1 L of 0.15 M oxalic acid. A Varian CP-3800 gas chromatography system (Varian Inc., Palo Alto, CA, USA) was used to measure total VFA and individual VFA (acetic, propionic, butyric, valeric, isobutyric and isovaleric acids) proportions following the method described previously [17]. For ammonia and LA analysis, the centrifuged liquid was diluted (1:40) in distilled water. Ammonia and L-LA and D-LA concentrations were determined using an ABX Horiba Pentra 400 chemistry analyser (Horiba-ABXDiagnostics, Kyoto, Japan).

The rumen pH of each rumen-cannulated cow was measured in-vivo for 48 consecutive hours on days 13 and 14 of each period using an indwelling rumen pH probe (Ionode Pty Ltd., Queensland, Australia). The data were logged at 1 min intervals for the duration of the 48 h using a data logger (Delta Omh HD 2105.2, Delta OMH S.r.l., Via Marconi 5, 35030 Caselle di Selvazzano, Italy). Average pH, minimum pH and the length of time below pH 5.5 and 6.0, and above pH 6.0 and 6.6 were calculated.

### 2.3. Grazing Swards

The experiment was undertaken in 2016 on swards sown in 2010, 2012 and 2013 as described by previously [9]. Briefly, swards sown in 2010 consisted of perennial ryegrass (*Lolium perenne* L.) only (cv. Tyrella sown at a rate of 29 kg/ha), and perennial ryegrass and white clover (Tyrella as above plus a 50:50 mixture of Chieftain and Crusader medium leaf clover cultivars sown at rate of 5 kg/ha, 50:50 mixture at 5 kg/ha). Swards sown in 2012 and 2013 were perennial ryegrass only (50:50 mixture of Astonenergy (tetraploid) and Tyrella (diploid) cultivars sown at a rate of 27.2 kg/ha), and perennial ryegrass and white clover (Astonenergy and Tyrella as above plus a 50:50 mixture of Chieftain and Crusader medium leaf clover cultivars sown at a rate of 5 kg/ha). A separate farmlet was created and fenced for each treatment. Fertiliser application to the swards was carried out from late January to mid-September in accordance with The Nitrates Directive (91/676/EEC). 

### 2.4. Grazing Management

The grazing treatments (Grass and Grass-Clover) were rotationally grazed for the duration of the experiment, achieving 8.3 rotations between mid-February and Mid-November. Fresh herbage was allocated daily, with a daily herbage allowance of 18 kg DM/cow, as described by Egan et al. [15]. Target post-grazing sward height for each treatment was 3.5 cm for the first and last rotations, and 4 cm in the other rotations. No concentrate or silage was fed during the experimental period. 

### 2.5. Total Mixed Ration Diet Treatment

The TMR group were housed year-round in cubicle accommodation. They were fed each morning using a Keenan Klassik 140 diet feeder (Keenan Ltd., Co., Carlow, Ireland) and were fed via a Griffith Elder feeding system (Griffith Elder Ltd., Suffolk, UK) which recorded daily intake. The TMR consisted of (kg DM) 7.15 grass silage, 7.15 maize silage and 8.3 concentrates. The concentrate blend consisted of (g/kg DM) 130 maize, 155 molassed beet pulp, 300 soybean meal, 120 maize distillers, 150 barley, 75 rapeseed meal, 30 megalac (Volac, Killeshandra, Co., Cavan, Ireland), 5 salt, 7 acid buffer (Celtic Sea Minerals, Co., Cork, Ireland), 28 minerals and vitamins. Samples of the three main TMR components were collected weekly and dried at 90 °C for 24 h to determine DM%. The fresh weight inclusion of forages was adjusted weekly to account for any variances which occurred in the DM% of the forages. Grass silage and maize silage samples were collected weekly for analysis. Grass silage was analysed using near infrared reflectance spectroscopy (NIRS) with a FOSS 6500 (FOSS Ireland Ltd., Dublin, Ireland) while maize silage was analysed by FBA Laboratories Ltd. (Co. Waterford, Ireland). Analysis included dry matter, ash, neutral detergent fibre (NDF), acid detergent fibre (ADF), starch (maize silage), crude protein and organic matter digestibility (OMD) (grass silage). Concentrates were sampled monthly and analysed using NIRS for ash, moisture, NDF, oil and crude protein.

### 2.6. Sward Measurements

Herbage representative of that selected by the grazing herd was collected in each paddock prior to grazing using a Gardena hand shears (Accu 60; Gardena International GmbH, Ulm, Germany). Herbage samples were frozen following collection. Samples were bowl-chopped (Muller, Type MKT 204 Special, Saabrücken, Germany) to give a homogenous sample length, freeze-dried and milled through a 1-mm screen.

Following processing, the selected herbage samples were analysed by wet chemistry for OMD, crude protein, NDF, ADF and ash contents. The OMD was estimated using the in-vitro neutral detergent cellulase method (FibertecTM Systems; Foss, Ballymount, Dublin, Ireland) as described previously [18]. Briefly, crude protein concentration was determined using a N analyser (FP-628; Leco Australia Pty Ltd., New South Wales, Australia) based on the AOAC method 990-03 (AOAC 1990). The NDF and ADF concentrations were determined using a fibre analyser (Ankom Technology, Macedon, NY, USA) based on the method described previously [19]. Amylase and a sodium sulphite solution were used in the NDF concentration determination process. The NDF and ADF values do not include ash. Ash concentration was estimated by burning a subsample in a muffle furnace at 500 °C for 12 h.

The sward white clover content was measured in each paddock immediately prior to grazing. Grab samples were taken diagonally across the paddock using a Gardena hand shears (as described above). The sample from each paddock was well mixed and two 70 g sub-samples were removed and separated into the grass and clover proportions. These samples were then dried at 90 °C for 16 h and the DM of each sample recorded to determine paddock clover content.

### 2.7. Animal Measurements

All milk production results reported in this paper are from the larger study [15] and are therefore the average milk production of a group of 17 cows which were grazing contemporaries of the rumen-cannulated cows. Milk production was quantified during the three seasons when rumen sampling occurred—spring (April/May), summer (June/July) and autumn (August/September). Milk yields (kg) were recorded daily at AM and PM milking for each individual cow using Dairymaster milk recorders (Dairymaster; Causeway, Co., Kerry, Ireland). Milk fat, protein, lactose and milk urea nitrogen (MUN) concentrations were determined using the Milkoscan 203 (DK-3400; Foss Electric, Hillerød, Denmark) from one successive evening (Tuesday) and morning (Wednesday) milk sample weekly for each animal. Dry matter intake (DMI) was estimated in each measurement season; spring, summer and autumn, using the *n*-alkane technique as described previously [9].

### 2.8. Statistical Analysis

Analyses were conducted using SAS (SAS Institute Inc., Cary, NC, USA) version 9.3. Data were checked for normality using PROC UNIVARIATE and analysed by linear mixed models that allowed for repeated measurements using PROC MIXED. 

Rumen data were analysed using the following model:

Y = u + Ti + (P)Sjk + A(T)il + T × (P (S)) ijk + e

where u = mean; Ti = treatment (i = 1 G, 2 GC, 3 TMR); Pj = period (j = 1…3); Sk = season (k = 1 spring, 2 summer, 3 autumn); A(T)il = the effect of animal within treatment (l = 1 … 3); T × (P(S) ijk = the interaction of treatment and period within season and e = residual error term.

The model included season as the repeated measure. Animals were grouped by treatment and included as the random effect. The fixed effects were treatment, period and season. The model specified the compound symmetry structure. 

Milk production data were analysed using the following model:

Y = u + Ti + Sj + A(T)ik + T × (S) ij + e

where u = mean; Ti = treatment (i = 1 G, 2 GC, 3 TMR); Sj = season (j = 1 spring, 2 summer, 3 autumn); A(T)ik = the effect of animal within treatment (l = 1 … 17); T × (S) ij = the interaction of treatment and season and e = residual error term.

The model included season as the repeated measure. Animal was the random effect. The fixed effects were treatment and season. The model specified the compound symmetry structure. 

Herbage data were analysed using the following model:

Y = u + Ti + (P)Sjk + T × (P (S)) ijk + e

where u = mean; Ti = treatment (i = 1 G, 2 GC, 3 TMR); Pj = period (j = 1…3); Sk = season (k = 1 spring, 2 summer, 3 autumn); T × (P(S)) ijk = the interaction of treatment and period within season and e = residual error term.

The model included season as the repeated measure. The model specified the compound symmetry structure. For all data, the Tukey–Kramer multiple range test was used for mean separation (*p* < 0.05). The regression procedure of SAS (2003, SAS Institute Inc., Cary, NC, USA) was used to determine the relationship between sward clover content and rumen ammonia concentration, between sward clover content and MU concentration, between rumen ammonia concentration and milk MU concentration, between herbage crude protein concentration and MU concentration, and between herbage crude protein content and rumen ammonia concentration.

## 3. Results

### 3.1. Herbage Chemical Composition

The crude protein concentration of the diet was significantly (*p* < 0.05) greater for the TMR treatment compared to Grass (Table 1). Diet crude protein concentration was lower in autumn and NDF was greater in autumn compared to spring and summer (Table 1). No significant treatment effects were detected for herbage OMD, ADF or ash content. 

### 3.2. Dry Matter Intake

The Grass-Clover cows had significantly greater DMI than the TMR cows in the spring (Table 2). All cows had a similar DMI in the summer while in the autumn the TMR cows had significantly greater DMI than both grazing groups. There was no significant difference between the grazing groups at any of the measurement periods. The DMI of the TMR cows increased significantly from spring to summer to autumn. The Grass-Clover cows had significantly lower DMI in the autumn compared to the spring and summer. The Grass cows had a similar DMI throughout lactation. 

### 3.3. Volatile Fatty Acids

There was a treatment × season interaction effect (*p* < 0.05) on total VFA concentration in both the AM and PM sampling times (Table 3 and Table 4). For the AM samples, in the spring and summer there was no difference among treatments, but in autumn the Grass-Clover cows had a significantly greater total VFA concentration than the TMR cows. For the PM samples, in spring the Grass-Clover cows had a higher total VFA concentration than the TMR cows. In summer, the Grass-Clover cows were higher than Grass cows and in autumn both Grass-Clover and Grass cows were higher than TMR for total VFA concentrations. For the Grass cows, total VFA concentrations were significantly greater in the Autumn PM samples than the summer PM samples. The Grass-Clover cows had significantly greater total VFA concentrations in the summer PM samples than the spring PM samples. On average, cows on the Grass-Clover treatment had a greater rumen VFA concentration than cows on both the Grass and TMR treatments (*p* < 0.05), (Table 3). The Grass treatment also resulted in a significantly greater rumen VFA concentration than that of the TMR treatment. 

There was a treatment × season interaction (*p* < 0.05) on acetic acid proportion in the rumen. In the AM Grass cows had significantly lower acetic acid in the spring compared to TMR-fed cows, but in summer and autumn there was no differences between the treatments (Table 3). In the PM, in spring TMR cows had a greater proportion of acetic acid in the rumen than both Grass-Clover and Grass cows, in summer all treatments had similar proportions of acetic acid, and in autumn TMR cows had a greater proportion than Grass-Clover cows (Table 4). For the AM samples the Grass cows had significantly greater proportion of acetic acid in the autumn than the spring and summer. In summary, as can be seen from Table 3 and Table 4, there was an overall treatment effect with TMR cows having greater (*p* < 0.05) acetic acid compared to both grazing groups.

There was a significant treatment effect on the proportion of rumen butyric acid in the PM with Grass cows having a greater proportion of butyric acid than TMR cows (Table 4). 

There was also a treatment × season interaction effect on the proportions of isobutyric and isovaleric acids. In both the AM and PM samples (Table 3 and Table 4, respectively), isobutyric acid proportions in the spring were the same amongst the three treatments, but in summer Grass-Clover was greater than Grass and TMR, while in autumn Grass-Clover was again the highest. The Grass-Clover treatment also had significantly greater isobutyric acid concentrations in the summer and autumn in comparison to the spring. The Grass-Clover treatment had the highest concentration of isovaleric acid in all three seasons in both AM and PM samples throughout the study (Table 3 and Table 4), being significantly greater than the Grass treatment in the summer sampling period. 

There was a treatment × season interaction (*p* < 0.05) on the proportion of valeric acid in the rumen. In spring, both the AM and PM samples had similar valeric acid across all three treatments (Table 3 and Table 4). In the summer and autumn, the Grass cows had significantly greater proportion of valeric acid than in the spring. 

While no significant treatment × season interaction for the proportion of propionic acid in the rumen was evident in the AM sampling (Table 3), there was an interaction in the PM samples (Table 4). In the spring the Grass treatment had significantly greater propionic compared to the TMR treatment. In the Autumn the Grass clover treatment had significantly greater propionic acid proportion than the TMR treatment. The proportion of propionic acid in the rumen of the Grass-Clover cows was similar across all three seasons, as was that of the TMR cows. 

The Grass-Clover treatment had the greatest concentration of L-lactic acid and D-lactic acid in the rumen, greater than that of the TMR and Grass treatments (*p* < 0.05), (Table 3 and Table 4). As can be seen from Table 5, diet had no effect on rumen pH. 

### 3.4. Rumen Ammonia and Milk Urea Nitrogen

There was a treatment × season interaction (*p* < 0.05) on rumen ammonia concentrations (Table 3 and Table 4). All treatments had similar rumen ammonia concentrations in the spring AM samples (Table 3). However, the Grass-Clover cows had significantly greater rumen ammonia concentrations than the Grass and TMR cows in the summer AM samples, and also than the TMR cows in the autumn AM samples. No difference was observed between the rumen ammonia concentrations of the Grass and TMR treatments in the spring, summer or autumn AM samples. 

For the PM samples (Table 4), the Grass-Clover cows had significantly greater rumen ammonia concentration than the TMR cows across all three seasons. The Grass-Clover cows had greater rumen ammonia than the Grass cows in the summer PM samples but both treatments had similar concentrations in the spring and autumn. The Grass cows had greater ammonia concentrations than the TMR cows in the autumn PM sampling but both treatments had similar concentrations in the spring and summer. 

While all three treatments had similar MU concentrations in the spring (Table 2), MU concentrations were greater in the milk produced by the Grass-Clover cows in the summer and autumn than the milk produced by both the Grass and TMR cows (*p* < 0.05). The milk from the Grass cows had a significantly greater MU concentration than the milk from the TMR cows in the autumn. The MU concentration increased for all three treatments as the lactation progressed, although this increase from spring to autumn was the smallest in the TMR treatment only increasing by 30%; in contrast the Grass-Clover treatment increased by 83% and the Grass treatment by 100%. 

The Grass-Clover cows had the greatest milk solids production in the spring (Table 2), significantly greater than the TMR cows. The TMR treatment had the greatest milk solids production in both summer and autumn, being significantly greater than Grass cows in summer and autumn but not in spring. The Grass-Clover and TMR treatments had similar milk solids production in the summer, but TMR had significantly greater milk solids yield in the autumn.

The TMR treatment had significantly lower milk protein content in the spring than both grazing treatments. All three treatments had similar milk protein contents in the summer and autumn. Milk protein content increased for all three treatments as lactation progressed with all three treatments having significantly greater milk protein content in the autumn than in the spring (Table 2). 

**Figure 1:**
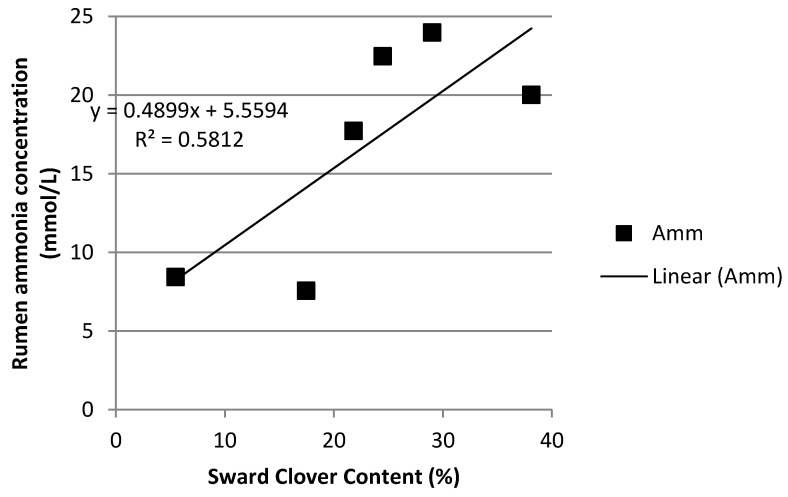
Relationship between sward clover content and rumen ammonia concentration.

**Figure 2:**
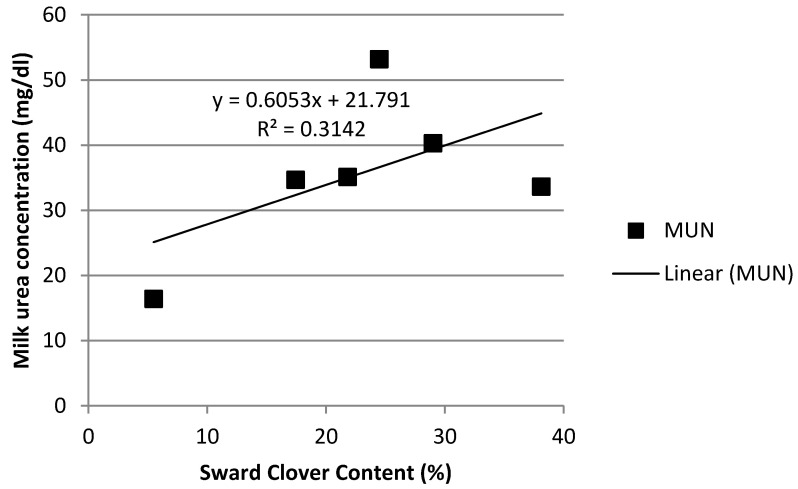
Relationship between sward clover content and milk urea nitrogen concentration.

**Figure 3:**
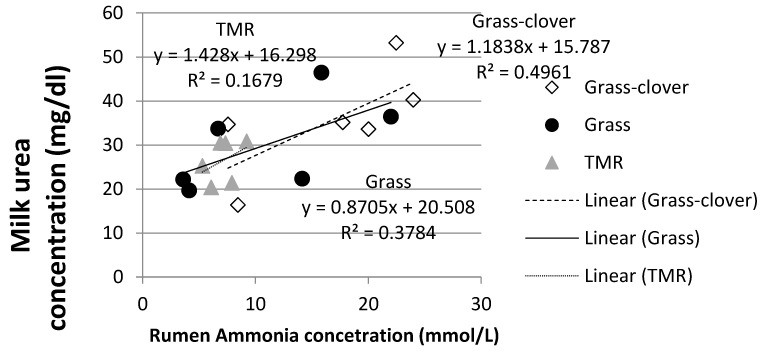
Relationship between rumen ammonia concentration and milk urea nitrogen concentration.

As can be seen in Figure 1, rumen ammonia concentrations in the Grass-Clover treatment increased as sward clover content increased. Clover content accounted for 58% of the variation in ammonia concentration. For every 1% increase in clover content, rumen ammonia concentration increased by 0.49 mmol/L. Similarly, as can be seen in Figure 2, clover content accounted for 31% of the variation in MU. Rumen ammonia concentration was significantly and positively associated with MU concentration (Figure 3) in all three treatments. 

**Figure 4:**
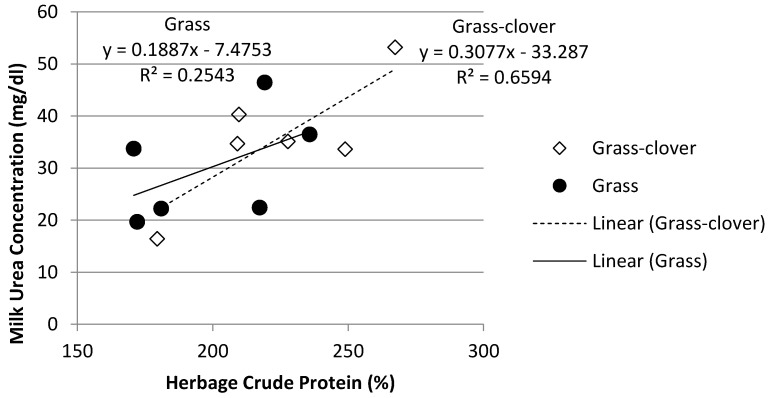
Relationship between herbage crude protein concentration and milk urea nitrogen concentration.

**Figure 5:**
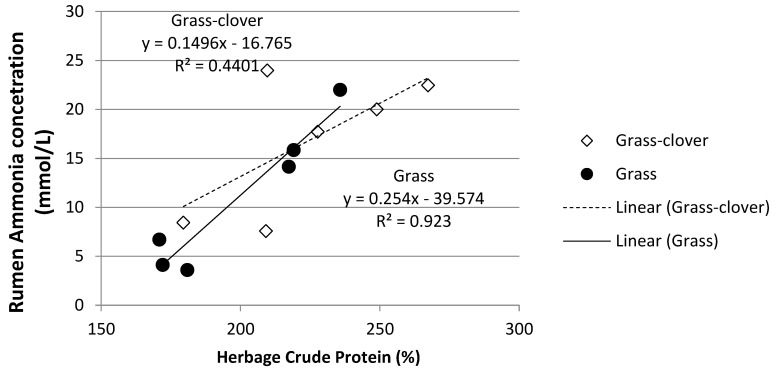
Relationship between herbage crude protein concentration and rumen ammonia concentration.

The MU concentration increased as herbage crude protein increased in both grazing treatments (Figure 4). Herbage crude protein content accounted for 66% of the variation in MU for the Grass-Clover treatment while only accounting for 25% of the variation in the Grass treatment. Similarly, there was a correlation between increasing herbage crude protein content and increasing rumen ammonia concentration (Figure 5). Herbage crude protein content accounted for 44% and 92% of the variation in rumen ammonia concentration in the Grass-Clover and Grass treatments, respectively. 

## 4. Discussion

Total rumen VFA concentration for the Grass treatment are within the range previously described [20,21], while total VFA concentration for the Grass-Clover cows are within the range previously reported for mixed swards [22]. 

The TMR cows, whose diet consisted of a large proportion of concentrate (~33%), had total VFA concentration similar to that reported previously [23]. Notably clover inclusion altered rumen function significantly compared to the Grass treatment. The Grass-Clover treatment had the greatest concentration of L-lactic acid and D-lactic acid in the rumen, significantly greater than that of the TMR and Grass treatments. Accumulation of lactic acid often occurs after engorgement of large quantities of soluble carbohydrates [24], indicating the superior quality of the grass-clover diet. The higher total VFA concentration of the Grass-Clover treatment compared to the Grass treatment is in line with results previously reported [12,14] who all showed higher rumen VFA concentrations in cattle when white clover was included as part of the diet. However, in the case of Dewhurst et al. [12], silages were used while Ribeiro Filho et al. [14] focused on two growth stages of the plant. 

We, however, looked at the differences among treatments by season. Both gazing treatments had a similar total VFA concentration in the spring probably because they had quite a similar plant content and structural profile as a result of low sward white clover content in the Grass-Clover treatment at this time. Total rumen VFA concentrations were higher for both the Grass and Grass-Clover treatments across all three seasons compared to TMR. While the VFA concentration of the Grass-Clover treatment increased as the year progressed (and clover content increased), the total VFA concentration of the Grass treatment declined in summer before increasing again in the autumn sampling period. This is most likely as a result of poorer grass quality in summer due to the plant entering its reproductive stage with greater fibre content and lower digestibility values [20]. As would be expected, given the consistency of the diet, total VFA concentrations for the TMR treatment remained constant throughout the year. The increased total VFA concentration of the Grass-Clover treatment in the summer and autumn can be attributed to improved sward quality due to increased clover content in the sward. Clover is a high quality, highly digestible plant and it improves the digestibility of the sward overall, particularly in summer during the reproductive growth phase of the perennial ryegrass plant and in the autumn when there is traditionally more dead material in the sward [25]. 

The lower NDF concentration, combined with higher crude protein value of the Grass-Clover treatment compared to the Grass treatment may contribute to more rapid and extensive breakdown of the rumen contents [26], leading to increased total VFA concentration. The higher total VFA concentration of the Grass-Clover cows compared to the TMR cows was unexpected as previous studies [27,28] showed increased total VFA concentration in high concentrate, high starch diets compared to a forage-only diet. However, these forage-only diets consisted mainly of grass, grass/silage combinations with no clover inclusion. It is difficult to attain a direct comparison from previous studies of grass clover and TMR diets. In addition, careful consideration should be paid to the quality of the forage. In those other studies [27,28] the quality of forage on offer was not as high as in the current study. The increased crude protein concentration and the reduced fibre content of grass-clover diets [29,30] could result in an increased passage rate of the Grass-Clover diet [19,31,32]. This could positively influence voluntary intake and animal performance [33]. However, clover inclusion did not increase intake in the current study. Therefore, it is most likely that clover inclusion resulted in increased digestibility of the diet, as previously reported [13,31,34]. 

The greater VFA concentration in the rumen fluid of the Grass-Clover cows compared to that of the Grass cows is similar to that found by Dewhurst et al. [12] who compared grass silages and legume silages for milk production. The increased VFA concentration observed may be a driver of the increased milk solids output from the Grass-Clover cows. 

The proportion of isobutyric and isovaleric acids in the rumen, both indicators of excess rumen degradable protein [35], were significantly greater in the Grass-Clover than the TMR treatment in the summer and autumn sampling periods. The Grass treatment also had a greater proportion of isobutyric acid in the summer and autumn than the TMR treatment, particularly in the PM samples. This is most likely a consequence of the excess protein available in the Grass and Grass-Clover diets. This agrees with the greater rumen ammonia concentration on the two grazing treatments compared to the TMR treatments. The crude protein concentration of well-fertilized, well-managed grass is usually in excess of the requirements of dairy cows [36]. Excess dietary crude protein is manifested in increased rumen ammonia concentrations, and the excess N is excreted by the cow and associated with an increase in urinary N concentration and excretion and with an increase in MUN [23,37]. This can have negative impacts on the environment through N loading on a small area through urinary N excretion. Urea equilibrates rapidly throughout body fluids, including milk, and the concentration of MUN is thought to reflect the concentration of blood urea N [38,39]. Therefore, MUN may serve as an index of inefficient N utilization in the lactating dairy cow [40]. 

In Ireland, herbage crude protein concentration can remain greater than 200 g/kg DM throughout the grazing season in well-managed, fertilised perennial-ryegrass dominant pastures [41,42] or mixed swards of perennial ryegrass and white clover [43]. Similarly, as can be seen in Figure 5, increased herbage crude protein concentration was associated with greater concentrations of rumen ammonia, which in turn was associated with an increased concentration of MUN. While rumen ammonia concentration increased as the year progressed for all three treatments, the increase was most pronounced in both grazing treatments, particularly in the Grass-Clover treatment. White clover swards tend to have higher crude protein content, increased excess of rumen degradable protein [12] and a faster rate of degradation in the rumen than grass-only swards [12,13]. As can be seen from Figure 1, Figure 2 and Figure 3, increasing the clover content in the diet was associated with increased rumen ammonia concentrations, which was associated with increased concentration of MUN. Figure 4 shows the correlation between increasing sward clover content and increasing herbage crude protein concentration, which resulted in increased rumen ammonia and MUN concentration. It has been reported that the provision of supplementary energy to high protein diets has been negatively associated with MUN content [44]. This may explain the reduced rumen ammonia and MUN concentration of the TMR treatment as energy supplementation in the form of concentrate feed, along with a lower crude protein intake, reduced rumen ammonia concentration and MUN. The lower rumen ammonia concentrations of the TMR cows are similar to those reported by Reis and Combs [45] who demonstrated more efficient use of rumen degradable protein in high concentrate treatments due to a lower input of dietary protein while also supplementing with high energy feeds to increase N use efficiency in the rumen. 

A high proportion of propionic acid in the rumen indicates a low fibre diet [46] and is associated with a high starch or highly digestible diet [47]. In the current study, the TMR cows whose diet consisted of 33% concentrate, 33% maize silage and 33% grass silage had a significantly lower proportion of propionate than that of the Grass-Clover and Grass treatments. Correspondingly, the TMR treatment also resulted in the highest proportion of acetic acid in the rumen. It has been shown that high forage diets stimulate higher rates of saliva production, better rumen buffering and greater acetate production which support increased milk fat concentration [48]. However, in the current study treatment, undertaken across one lactation, had no effect on milk fat concentration and there was no evidence of milk fat depression. This indicates that there was sufficient dietary fibre on all three treatments. It also probably reflects the fact that there were no significant differences in rumen pH among treatments.

## 5. Conclusions

Rumen function and animal performance were significantly influenced by dairy cow diet. Including white clover in grazed grass swards resulted in increased milk production compared to Grass, similar milk solids production to TMR, and greater total rumen VFA concentration compared to both the Grass and TMR treatments. However, the increased rumen ammonia and MUN concentrations on the Grass-Clover treatment may be a cause of concern from an environmental perspective. Therefore, while recent studies have shown the benefits of clover inclusion in terms of increased milk production in pasture-based systems, further research is required into the wider consequences in terms of its potential environmental sequelae. Future research should also place these results in the context of life cycle assessment models in order to provide a holistic comparison of the systems from a carbon footprint perspective. 

## Figures and Tables

**Table 1:** Effect of treatment (Grass receiving 250 kg N/ha/year—G; Grass-Clover receiving 250 kg N/ha/year—GC; total mixed ration diet—TMR) and season (Spring: April May, Summer: June July, Autumn: August September) on herbage chemical composition in 2016.

	Spring			Summer			Autumn			S.E.	Treatment	Season	Treatment × Season
	G	GC	TMR	G	GC	TMR	G	GC	TMR				
Crude Protein (g/kg DM)	176.5	194.3	159.1	194.1	238.3	159.1	227.5	226.4	159.1	12.65	<0.05	<0.05	NS
OMD (g/kg DM)	807.3	859.6	790.0	763.1	791.7	790.0	695.9	788.4	790.0	32.21	NS	NS	NS
ADF (g/kg DM)	220.6	195.8	223.2	223.7	227.7	223.2	233.9	227.1	223.2	12.63	NS	NS	NS
NDF (g/kg DM)	349.1	302.6	345.7	369.1	352.0	345.7	414.7	385.0	345.7	15.58	NS	<0.05	NS
Ash (g/kg DM)	73.8	79.6	118.2	82.9	85.3	118.0	83.5	97.0	118.0	8.20	<0.05	NS	NS

S.E., Standard Error; NS, Not Significant: *p* > 0.05.

**Table 2:** Effect of treatment (Grass receiving 250 kg N/ha/year—G; Grass-Clover receiving 250 kg N/ha/year—GC; total mixed ration diet—TMR) and season (Spring: April May, Summer: June July, Autumn: August September) on daily milk yield, daily milk solids yield, milk protein, fat, lactose content, milk urea nitrogen content and daily dry matter intake.

	Spring			Summer			Autumn			S.E.	Treatment	Season	Treatment × Season
	G	GC	TMR	G	GC	TMR	G	GC	TMR				
Daily milk yield (kg/cow)	26.7 ^abc^	28.6 ^a^	26.8 ^ab^	20.9 ^def^	23.9 ^bcde^	24.4 ^cd^	15.9 ^g^	18.5 ^fg^	21.6 ^ef^	0.99	<0.05	<0.001	<0.001
Daily milk solids yield (kg/cow)	2.0 ^ab^	2.1 ^a^	1.9 ^bc^	1.6 ^cd^	1.8 ^bc^	1.9 ^ab^	1.3 ^e^	1.5 ^de^	1.8 ^bc^	0.07	<0.05	<0.001	<0.001
Protein (g/kg)	33.4 ^ad^	33.9 ^abc^	30.2 ^h^	34.9 ^bcef^	34.9 ^def^	33.9 ^abde^	38.9 ^g^	37.6 ^g^	36.3 ^cfg^	0.75	<0.05	<0.001	<0.001
Fat (g/kg)	39.1 ^ac^	41.7 ^abcd^	38.5 ^ad^	43.2 ^bde^	42.0 ^abcd^	44.9 ^bce^	46.1 ^be^	46.9 ^e^	47.0 ^be^	1.70	N.S	<0.001	<0.05
Lactose (g/kg)	48.8 ^a^	48.6 ^ab^	47.7 ^abd^	47.5 ^b^	47.8 ^ab^	47.7 ^ab^	45.3 ^c^	46.1 ^cd^	45.9 ^c^	0.39	N.S	<0.001	<0.001
Milk urea nitrogen (mg/dL)	20.5 ^a^	25.5 ^ac^	22.7 ^ad^	27.6 ^dc^	34.3 ^bg^	26.1 ^dc^	41.1 ^e^	46.7 ^f^	29.5 ^cg^	1.16	<0.001	<0.001	<0.001
Dry matter intake (kg/day)	15.7 ^acd^	18.5 ^ab^	15.6 ^c^	17.5 ^acd^	18.5 ^ab^	19.5 ^ad^	15.6 ^acd^	15.8 ^cd^	22.1 ^b^	0.87	<0.05	<0.001	<0.001

^a–h^ means within a row without a common superscript differ (*p* < 0.05), S.E., standard error; NS, not significant: *p* > 0.05.

**Table 3:** Effect of treatment (Grass receiving 250 kg N/ha/year—G; Grass-Clover receiving 250 kg N/ha/year—GC; total mixed ration diet—TMR) and season (Spring: April May, Summer: June July, Autumn: August September) on rumen total volatile fatty acid concentration, individual volatile fatty acid proportions, L-lactic and D-lactic acid concentration, and ammonia concentration (AM sampling).

AM													
	Spring			Summer			Autumn			S.E.	Treatment	Season	Treatment × Season
	G	GC	TMR	G	GC	TMR	G	GC	TMR				
Total VFA (mmol/L)	123 ^abc^	122 ^ab^	121 ^ab^	126 ^abc^	137 ^c^	122 ^abc^	124 ^abc^	135 ^ac^	111 ^b^	4.0	<0.001	<0.05	<0.05
Acetic Acid (%)	65.4 ^a^	67.5 ^abc^	68.3 ^bc^	67.4 ^abc^	66.2 ^ab^	67.7 ^abc^	69.0 ^c^	67.3 ^abc^	68.5 ^bc^	0.60	NS	<0.01	<0.01
Propionic Acid (%)	19.2	18.0	17.5	17.7	17.7	17.9	16.9	17.8	16.8	0.52	<0.05	<0.05	NS
Butyric Acid (%)	11.9 ^a^	11.2 ^a^	10.5 ^b^	11.7 ^a^	11.9 ^a^	10.5 ^b^	10.6 ^a^	11.0 ^a^	11.5 ^a^	0.41	NS	NS	<0.05
Isobutyric Acid (%)	0.9 ^a^	0.9 ^a^	1.0 ^ac^	0.9 ^a^	1.2 ^bc^	0.9 ^a^	1.0 ^a^	1.2 ^b^	1.0 ^abc^	0.04	<0.05	<0.001	<0.001
Valeric Acid (%)	1.6 ^a^	1.2 ^ab^	1.0 ^b^	1.1 ^b^	1.3 ^ab^	1.1 ^ab^	0.9 ^b^	1.2 ^ab^	1.0 ^b^	0.12	<0.05	NS	<0.001
Isovaleric Acid (%)	1.3 ^a^	1.3 ^a^	1.5 ^abc^	1.3 ^a^	1.8 ^bc^	1.5 ^ac^	1.4 ^a^	1.8 ^b^	1.5 ^abc^	0.08	<0.05	<0.05	<0.001
D-Lactic Acid (mmol/L)	0.4	1.2	0.9	1.0	1.3	1.1	0.8	1.3	1.0	0.19	<0.001	NS	NS
L-Lactic Acid (mmol/L)	0.2	0.5	0.4	0.5	0.6	0.5	0.3	0.5	0.4	0.63	<0.05	<0.05	NS
Ammonia (mmol/L)	3.9 ^a^	4.1 ^a^	8.4 ^abc^	9.1 ^bc^	15.8 ^d^	5.5 ^ab^	11.6 ^cd^	16.1 ^d^	8.1 ^abc^	1.01	<0.001	<0.001	<0.001

^a–d^ means within a row without a common superscript differ (*p* < 0.05), S.E., standard error; NS, not significant: *p* > 0.05.

**Table 4:** Effect of treatment (Grass receiving 250 kg N/ha/year—G; Grass-Clover receiving 250 kg N/ha/year—GC; total mixed ration diet TMR) and season (Spring: April May, Summer: June July, Autumn: August September) on rumen total volatile fatty acid concentration, individual volatile fatty acid proportions, L-lactic and D-lactic acid concentration, and ammonia concentration (PM sampling).

PM													
	Spring			Summer			Autumn			S.E.	Treatment	Season	Treatment × Season
	G	GC	TMR	G	GC	TMR	G	GC	TMR				
Total VFA (mmol/L)	145 ^abcd^	146 ^abc^	123 ^de^	128 ^cde^	153 ^ab^	132 ^bcde^	152 ^ab^	164 ^a^	121 ^e^	5.3	<0.001	<0.05	<0.05
Acetic Acid (%)	60.9 ^a^	63.1 ^a^	66.0 ^b^	62.3 ^a^	61.1 ^a^	63.3 ^ab^	63.6 ^ab^	63.3 ^a^	66.2 ^b^	0.59	<0.001	<0.001	<0.05
Propionic Acid (%)	21.7 ^b^	20.4 ^ab^	18.7 ^ac^	19.8 ^abc^	19.3 ^ac^	19.0 ^ac^	19.0 ^ac^	19.9 ^ab^	17.8 ^c^	0.46	<0.05	<0.05	<0.05
Butyric Acid (%)	14.6	13.3	12.3	14.5	15.0	13.8	12.8	12.1	12.5	0.44	<0.05	<0.001	NS
Isobutyric Acid (%)	0.6 ^a^	0.8 ^abc^	0.7 ^ac^	1.0 ^be^	1.2 ^d^	0.8 ^abc^	1.1 ^de^	1.2 ^d^	0.8 ^bc^	0.05	<0.001	<0.001	<0.05
Valeric Acid (%)	1.5 ^a^	1.2 ^ab^	1.4 ^b^	1.2 ^b^	1.6 ^ab^	1.1 ^ab^	1.3 ^b^	1.1 ^ab^	1.2 ^b^	0.09	<0.05	NS	<0.001
Isovaleric Acid (%)	1.0 ^a^	1.3 ^abc^	1.2 ^ab^	1.4 ^bc^	1.9 ^d^	1.3 ^abc^	1.7 ^bcd^	2.0 ^d^	1.7 ^cd^	0.06	<0.001	<0.001	<0.05
D-Lactic Acid (mmol/L)	0.5	0.8	0.6	0.4	0.7	0.4	0.8	0.9	0.8	0.15	NS	NS	NS
L-Lactic Acid (mmol/L)	0.2	0.4	0.3	0.3	0.4	0.2	0.3	0.4	0.3	0.06	<0.05	NS	NS
Ammonia (mmol/L)	4.5 ^ab^	11.2 ^bc^	3.2 ^a^	11.9 ^c^	21.9 ^d^	9.5 ^abc^	25.6 ^de^	30.4 ^e^	9.0 ^abc^	1.63	<0.001	<0.001	<0.001

^a–e^ means within a row without a common superscript differ (*p* < 0.05), S.E., standard error; NS, not significant: *p* > 0.05.

**Table 5:** Effect of treatment (Grass receiving 250 kg N/ha/year—G; Grass-Clover receiving 250 kg N/ha/year—GC; total mixed ration diet—TMR) and season (Spring: April May, Summer: June July, Autumn: August September) on rumen pH.

	Spring			Summer			Autumn			S.E.	Treatment	Season	Treatment × Season
pH	G	GC	TMR	G	GC	TMR	G	GC	TMR				
Time spent below pH 5.5	0.03	0.03	0.11	0.02	0.03	0.14	0.03	0.02	0.03	0.058	NS	NS	NS
Time spent below pH 6.0	0.21	0.39	0.30	0.29	0.20	0.27	0.33	0.24	0.26	0.106	NS	NS	NS
Time spent above pH 6.0	0.79	0.61	0.70	0.71	0.79	0.73	0.67	0.76	0.73	0.106	NS	NS	NS
Time spent above pH 6.2	0.63	0.36	0.51	0.50	0.57	0.42	0.61	0.53	0.42	0.111	NS	NS	NS

S.E., Standard Error; NS, Not Significant: *p* > 0.05.

## Data Availability

The data presented in this study are available on request from the corresponding author.
